# Chemical Enhancement for Retrofitting Moving Bed Biofilm and Integrated Fixed Film Activated Sludge Systems into Membrane Bioreactors

**DOI:** 10.3390/membranes9100135

**Published:** 2019-10-22

**Authors:** Zakhar Maletskyi, Dawit K. Zigta, Olga Kulesha, Harsha Ratnaweera

**Affiliations:** 1 Faculty of Science and Technology, Norwegian University of Life Sciences (NMBU), 1433 Aas, Norway; olga.kulesha@nmbu.no (O.K.); harsha.ratnaweera@nmbu.no (H.R.); 2 Institute of Water and Environment, Mekelle University, Mekelle 231, Tigray, Ethiopia; dkzigta@gmail.com; 3 Faculty of Chemical Technology, National Technical University of Ukraine “Igor Sikorsky Kyiv Polytechnic Institute”, 03056 Kyiv, Ukraine

**Keywords:** biofilm membrane bioreactor, chemical enhancement, coagulant, membrane fouling

## Abstract

Positive effects of retrofitting MBBR and IFAS systems into MBRs can be exploited by introducing chemical enhancement applying coagulants in the membrane separation step. The current study reports basic principles of chemical enhancement with aluminium sulphate coagulant in biofilm-MBR (Bf-MBR) based on results of total recycle tests performed at different dosages of the chemical enhancer and properties characterization of filtrates, supernatants and sediments. It demonstrates a possibility to achieve lower membrane fouling rates with dosing of aluminium sulphate coagulant into MBBR and IFAS mixed liquors by extending operational cycles by 20 and 80 time respectively as well as increasing operating permeability of membrane separation by 1.3 times for IFAS. It has been found that charge neutralization is the dominating mechanism of aluminium sulphate action as a chemical enhancer in Bf-MBR, however, properties of the membrane surface influencing charge repulsion of foulants should be considered together with the secondary ability of the coagulant to improve consolidation of sediments.

## 1. Introduction

Higher treatment standards [1] and growing needs for water reuse [2] are increasing the number of wastewater treatment plants suffering from an inadequate level of treatment and challenges of capacity increase. In order to meet the new challenges, the wastewater industry adopted two disruptive technological advances in biological wastewater treatment: Introduction of biofilm processes and membrane separation techniques [3,4]. The latter played an essential role in the development of on-site wastewater treatment [5] and stimulated a paradigm shift to the regenerative sanitation [6].

Comparing with conventional Activated Sludge (AS), biofilm processes are simpler in operation, have higher biomass activity and resistance against toxic substances [7]. In the Moving Bed Biofilm Reactor (MBBR), freely moving plastic media is applied as housing for biofilm, providing usage of the whole reactor volume and eliminating the need for biomass recirculation. MBBR and AS can be further combined into Integrated Fixed-film Activated Sludge (IFAS) process, where solids retention time (SRT) for full removal of organic matter is considerably shorter than in AS or MBBR individually [8].

With the introduction of the membrane separation techniques, conventional AS processes can be converted into Membrane Bioreactors (MBRs). Since membrane filtration allows higher sludge concentrations, volume of the activated sludge tank can be significantly reduced. In combination with the option to convert the secondary clarifier to an additional activated sludge tank, the treatment capacity of the existing plant can be significantly extended, securing at the same time better quality and stability of the effluent [3]. However, high operating costs associated with the energy demand and relative process complexity limit faster adoption of MBR as the preferred process over competing wastewater treatment technologies. From the operational point of view, membrane fouling remains the main technical challenge, followed by screening/pre-treatment, membrane aerator clogging and overloading.

Anticipating synergism between biological treatment in biofilms and separation of particles by membranes, the processes were evolutionary merged into a Biofilm Membrane Bioreactor (Bf-MBR). Such systems have outstanding flexibility in process design and configurations [9], decouple biological and particle separation processes, provide lower footprint and potential of membrane operation at higher fluxes, lower membrane fouling as well as offer stable operation under high organic loading [10].

Currently, Bf-MBR is still in the research phase [11], however successful applications are foreseen on cruise ships, mobile bases and in emergency management scenarios, for treatment of household saline wastewater [12]. In addition to new plants, existing MBBR plants can be relatively easy to convert into Bf-MBR, and AS plants can be rehabilitated, achieving compliance with tightening requirements on the removal of organic matter [13].

From another hand, Bf-MBR emerged as an alternative strategy to reduce the negative effects of membrane fouling by high biomass concentrations, particularly under low loading rates [14]. It is reasonable to expect that the implementation of attached biofilm can enhance filtration performance and reduce membrane fouling [11]. Wang et al. reported that the attached biomass decreased total filtration resistance by 48% and prolonged three-time operational cycles [15].

According to the study that adopted Hermia pore blocking models to assess the membrane fouling in BF-MBR [16], the primary mechanism is cake layer formation. However, sustainable operation of Bf-MBR correlated to the concentration of the submicron particle size fraction. At low MLSS concentration, a dense and low-porous cake layer with high resistance was formed, while high MLSS led to the formation of a dynamic cake layer, which had a low fouling resistance [17].

Contrary to those findings, worse membrane performance after addition of MBBR carriers has been shown [18] and explained by the overgrowth of filamentous bacteria that resulted in higher values of Extracellular Polymeric Substances (EPSs) that alter the surface properties of flocs and affect their deposition on the membrane surface. The presence of attached biofilm in Bf-MBR can result in floc breakage and produce smaller flocs, whose size is more similar to membrane pore size [19]. From another hand, larger carriers and lower filling fractions can flocculate suspended biomass and promote the formation of larger flocs, then reducing fouling rates [15].

There are studies reporting reduction of SMPs in Bf-MBR due to the ability of the biofilm to adsorb and bind those [15]. From another hand, some studies are reporting the opposite—higher content of proteins and polysaccharides due to an overgrowth of filamentous bacteria [18]. Therefore, membrane fouling remains the primary constraint for long-term sustainable operation of Bf-MBRs and a significant research challenge.

Since the effects of the attached biofilm and scouring between carriers and membrane on membrane fouling are not univocal, several membrane fouling control strategies were developed for Bf-MBR [20] including aeration [21], scouring effects of carriers [22], addition of adsorbents [23] and chemicals [24]. Coagulation has been used in MBR [25,26], and Bf-MBR [27,28], to improve membrane separation performances. Documented advantages of coagulation as applied in MBR are lower fouling rates, longer operational cycles and higher operating fluxes. Such effects were associated in the literature with the ability of coagulants to reduce soluble organic content of SMP/EPS [29], enlarge floc sizes [30], and increase cake porosity [31]. The main disadvantages of this approach are the consumption of additional chemicals and the necessity to introduce dosing control measures.

The best improvement in membrane performance was observed [10] for the higher dosage range using iron chloride. The improved membrane performances observed at the higher iron dosage was associated to the formation of lighter flocs with large surface areas which potentially can form a protective cake layer on the membrane surface thereby reducing fouling by the submicron fractions.

From another hand, [28] found that in contrast to the non-prepolymerized coagulants, prepolymerized aluminium coagulants were much more efficient for flux enhancement. They demonstrated the greatest fouling mitigation extent. The prepolymerized aluminium chloride of medium basicity had the highest bearing positive charge and demonstrated the greatest extent of fouling alleviation, which suggests the significance of the adsorption/charge neutralization mechanism in the flux enhancement in biofilm membrane bioreactor, while in case of non-prepolymerized iron (III) or aluminium sulphate the combination of the dissolved organic matter removal with the increase of floc relative hydrophobicity or the increase in particle size were not enough. To date, the investigation on the effects of operating conditions on membrane fouling in Bf-MBR is still limited, and studies focused only on the effects of HRT/SRT, aeration rate and bio-carriers filling ratio [32], while few and contradictory information is available on the effects of membrane flux enhancers [28].

Therefore, the objective of this study was to evaluate treatment efficiency and membrane fouling mitigation properties of aluminium sulphate coagulant as a chemical enhancer in two configurations of the Biofilm Membrane Bioreactor based on Moving Bed Biofilm (MBBR) and Integrated Fixed-film Activated Sludge (IFAS) processes, approaching operating conditions of real wastewater treatment systems.

## 2. Materials and Methods

Effects of chemical enhancement were studied applying aluminium sulphate coagulant in laboratory-scale membrane filtration unit, using two types of Mixed Liquor (ML) sampled from the Moving Bed Biofilm (MBBR) and Integrated Fixed-film Activated Sludge (IFAS) bioreactors.

### 2.1. Mixed Liquors

The MBBR was operated as a part of the Bekkelaget municipal wastewater treatment (BEVAS—Oslo, Norway), currently serving at the capacity of 300,000 pe (50 Mm^3^/year). The Municipality of Oslo has a plan to increase the capacity of BEVAS reaching 500,000 pe in 2040 [33], therefore retrofitting the plant to the Biofilm Membrane Bioreactor (Bf-MBR) with chemical enhancement is one of the possibilities to be explored. BEVAS treats typical municipal wastewater from the combined sewer with minor contributions of industrial wastewater. The raw wastewater composition can be described by average values of COD 750 mg/L, BOD 350 mg/L, N-total 60 mg/L, P-total 15 mg/L, TSS 400 mg/L [34]. The plant has sand traps and grids and primary sedimentation before the MBBR that provides removal of organic matter, followed by the secondary sedimentation with coagulant dosing. For this study case, the ML was sampled after MBBR and coagulation with polyaluminium chloride coagulant PAX-18 (Kemira, Espoo, Finland) at a dose of 0.7 mM-Al/g-SS.

The IFAS bioreactor was operated as a part of the onsite wastewater treatment plant with the capacity of 0.3 m^3^/d, serving an area of private houses (Aas, Norway) with a source-separated sewer [35]. Blackwater (BW) and greywater (GW) from the sewer network were supplied to the equalization tank of the system at the ratio BW:GW = 1:9. The raw wastewater composition varied by COD 142–262 mg/L, BOD 70–142 mg/L, P-total 8–14 mg/L, TSS 0.4–1.3 g/L. The biological treatment was operated for organic matter removal at the solid’s retention time of 20 days with recirculation of excess activated sludge. For this study case, the ML was sampled after the biological reactor without additional treatment.

Samples of Mixed Liquors were analysed following standard procedures [36], and results are reported in Table 1.

### 2.2. Experimental Setup

The membrane filtration experiments were conducted according to the modified Total Recycle Test (TRT) protocol [28]. Microporous (0.1 µm) flat-plate silica carbide membrane (Cembrane, Lynge, Denmark) was used for the studies with active surface area 0.043452 m^2^. Aluminium sulphate coagulant (4.3% Al), commercially available under ALS trademark (Kemira, Espoo, Finland), was used as a chemical enhancer.

During the tests, a known volume of chemical enhancer (470–3300 μL) was introduced to the constant volume of ML (2.2 L) using micropipette, simulating the time and mixing conditions at a wastewater treatment plant (30 sec rapid mixing and 90 sec slow mixing) by adjusting aeration in the filtration container. A dose of chemical enhancer can be adjusted by changing the additive volume.

Membrane filtration was carried out at constant flux (50 L/m^2^⋅h) provided by a peristaltic metering pump (Watson Marlow Qdos 30, Marlow, UK) with full recycling of the obtained filtrate and continuous recording of the transmembrane pressure (TMP) with a precise digital pressure transducer (Klay 8000, Dwingeloo, Nederland) until TMP drops by factor 1.5. Following filtration, the membrane was backwashed with deionized water at 500–600 L/m^2^⋅h and 1–3 bar. Membrane filtration experiments were conducted at the same temperature (18 °C) to exclude the influence of temperature on membrane permeability.

**Figure 1:**
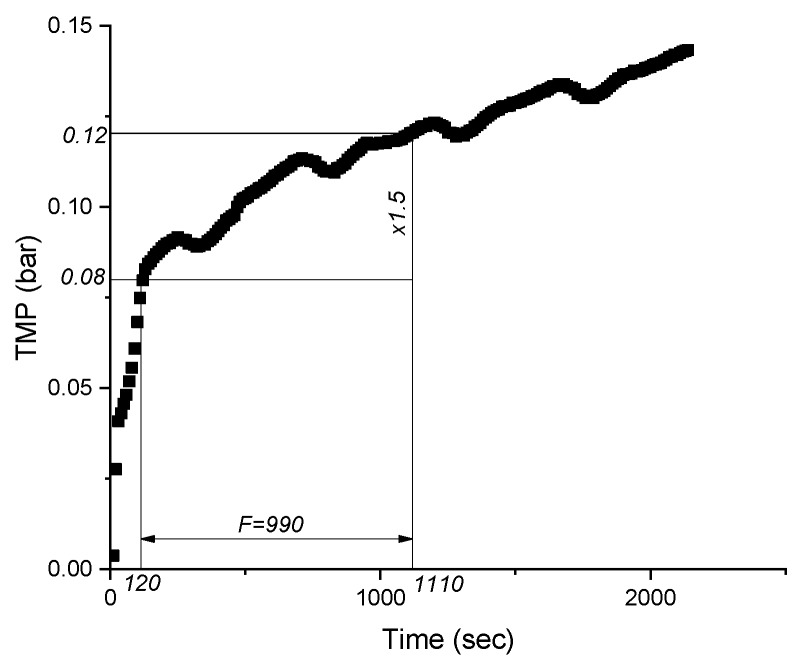
Identification of filtration duration (F) in the Total Recycle Test for the raw MBBR ML.

Operational curves of TMP as a function of time and respective filtered volume were used to calculate filtration duration (Figure 1), while supernatant and sediment were sampled and analysed within 30 min after the tests.

### 2.3. Analytical Techniques

Turbidity, zeta potential and concentration of orthophosphates were measured in supernatants and membrane filtrates after TRTs following standard procedures [37].

Samples of supernatants were filtered before turbidity and zeta potential measurements through the quantitative cellulose filter paper with the pore size 8–12 µm (Grade MN 640 md, Macherey-Nagel™, MACHEREY-NAGEL GmbH & Co. KG, Düren, Germany).

Zetasizer Nano-Z (Malvern, Malvern, UK) was used for zeta potential measurements. HACH 2100 N IS Turbidimeter was used for turbidity measurements (ISO 7027 method). The concentration of orthophosphates was measured using EasyChem Plus colourimetric analyser (SysteaTM, Systea S.p.A., Anagni, Italy) by following the automated method (USEPA Method 365.1).

Samples of sediments were tested for Capillary Suction Time (CST) using T304 test cell (Triton Ltd, Essex, UK). Microscopic images of sediments were taken at ×90 magnification with Leica DM 6B light microscope (equipped with Leica DMC4500 camera) and used to identify particle size distribution (PSD). Area of 2544 × 1816 pix was analysed for each image using ImageJ software package, identifying particle areas and converting into diameters that were used for the cumulative distribution plots according to the [38]. The PSD results are expressed according to the [39] as distribution points, mean values, span and uniformity.

### 2.4. Data Processing

Partial Least Squares regression was applied to investigate relationships between the dose of the coagulant, parameters of MLs and the fouling indicators in the Total Recycle Test. One of the main advantages of PLS is the removal of noise and collinearity between the input variables [40]. The Unscrambler^®^ X10.3 (CAMO Software AS, Oslo, Norway) was used for data analysis. Parameters included in the modelling are shown in the Table 2.

Average permeability was calculated for every test following (1):

(1) $$P_{\mathrm{av}}=\mathrm{Average}\left(\frac{\sum P_{i}^{in}}{N};\;\frac{\sum P_{m}^{fin}}{N}\right),$$

where N is the number of values taken into consideration, N = 10; $\frac{\sum P_{i}^{in}}{N}$—the average of the initial normalized permeability values—first ten values, excluding the ramp of the peristaltic pump (seconds no. 200–210); $\frac{\sum P_{n}^{fin}}{N}$—the average of the final permeability values.

**Figure 2:**
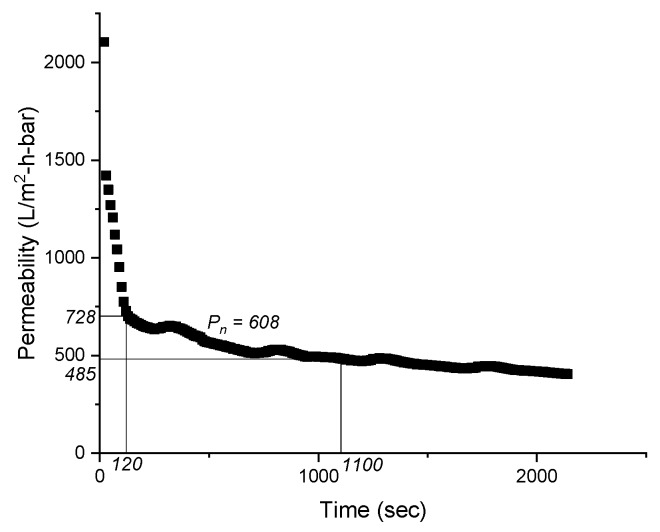
Determination of average permeability (P_n_) in the Total Recycle Test for the raw MBBR ML.

For this purpose, a steep phase of the development of permeability overtime was chosen (Figure 2) to cover the initial rapid flux decline phase (conditioning fouling), which is characterized by pore blocking and adsorption of the SMP on the membrane [41].

## 3. Results

### 3.1. Membrane Filtration Performance

Dosing of coagulant into mixed liquors improves membrane filtration performance in terms of filtration duration for both MBBR and IFAS. However, different trends are observed for membrane permeability: declining for MBBR and slightly increasing for IFAS.

**Figure 3:**
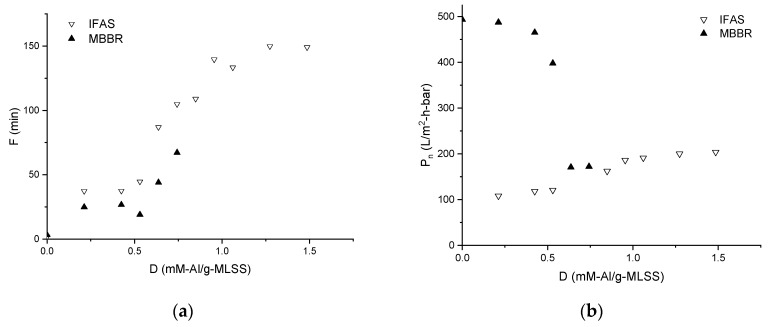

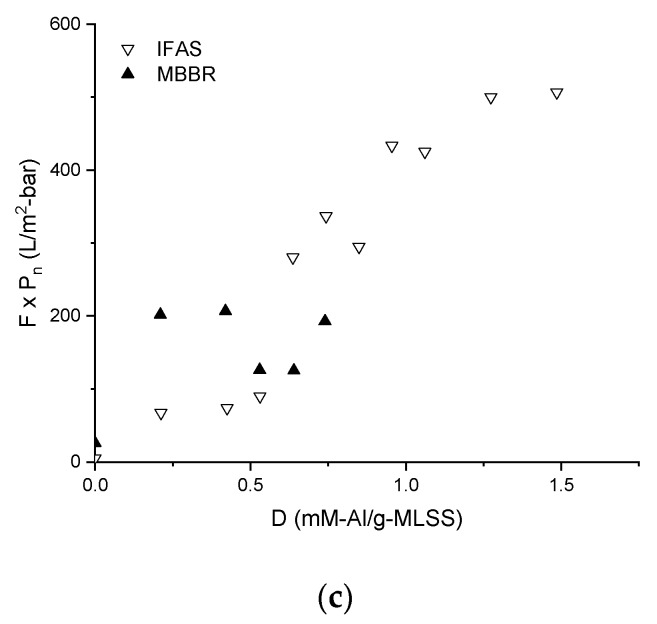
Influence of the chemical enhancer dose on filtration duration F (**a**), membrane permeability P_n_ (**b**) and their product F × P_n_ (**c**) during filtration of Mixed Liquors from MBBR and IFAS.

As follows from the Figure 3a, dosing of coagulant up to 0.5 mM-Al/g-MLSS moderately increases filtration duration to 45 min for IFAS and 25 min for MBBR from initial 2–3 min for both MLs. Further increase of dosing leads to the steep incline of the filtration duration curves. At the same time, membrane permeability (Figure 3b) demonstrates steep decline from 500 to 170 L/m^2^⋅h⋅bar at the dose >0.5 mM-Al/g-MLSS for MBBR, while relatively stable values for IFAS: 100–120 L/m^2^⋅h⋅bar at the dose <0.5 mM-Al/g-MLSS and 200 L/m^2^⋅h⋅bar at higher doses. The effect of the two values can be considered as the product of filtration duration and permeability (Figure 3c): Below 200 L/m^2^⋅bar for MBBR and continuously increasing for IFAS up to 500 L/m^2^⋅bar.

### 3.2. Properties of the Supernatants and Filtrates

**Figure 4:**
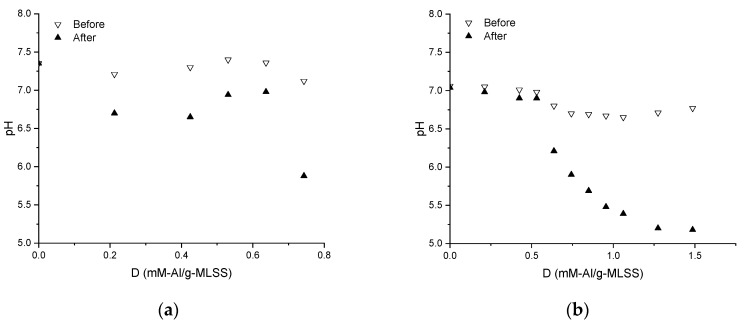
Influence of chemical enhancer dose on supernatant pH after dosing into Mixed Liquors from MBBR (**a**) and IFAS (**b**).

Filtrates obtained during TRTs were of stable quality by turbidity (<0.3 NTU), suspended solids (<0.01 g/L) and COD (<30 mg/L). The pH drop after dosing of coagulant was observed for both MLs (Figure 4), but more pronounced for IFAS, from 7.0 to 5.2.

**Figure 5:**
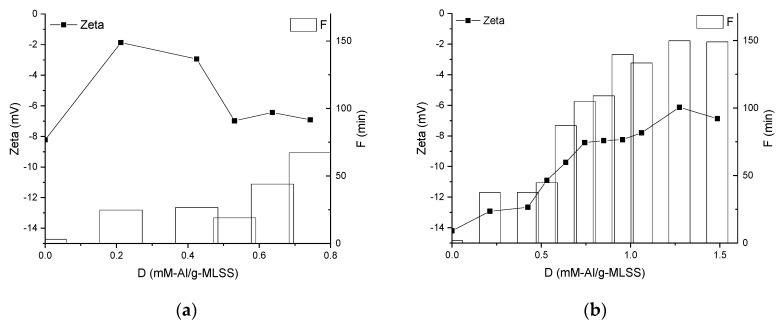
Influence of chemical enhancer dose on supernatant electrokinetic potential (zeta) and filtration duration of Mixed Liquors from MBBR (**a**) and IFAS (**b**).

The different character of the electrokinetic potential change was observed for MBBR and IFAS MLs during dosing of the coagulant (Figure 5). It was increasing for MBBR reaching the maximum of −2 mV at 0.2 mM-Al/g-MLSS and after that decreasing almost to initial value. The continuous increase of the zeta-potential was observed for IFAS ML with dosing of the coagulant from −14 to 7 mV. There is a clear correlation between zeta-potential and filtration duration for IFAS (Figure 5b).

**Figure 6:**
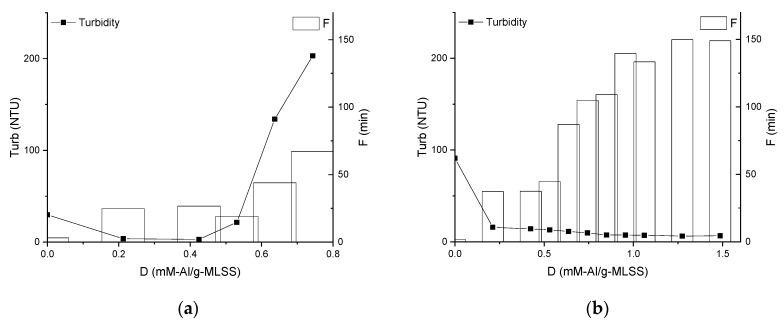
Influence of chemical enhancer dose on supernatant turbidity and filtration duration of Mixed Liquors from MBBR (**a**) and IFAS (**b**).

Supernatant turbidity was observed raising for MBBR and declining to a low level in IFAS. While turbidity is increasing in the MBBR ML and keeping constant at a relatively low level (<15 NTU) in the IFAS ML at dosages >0.4 mM-Al/mg-MLSS, the filtration duration continues raising in both cases (Figure 6).

**Figure 7:**
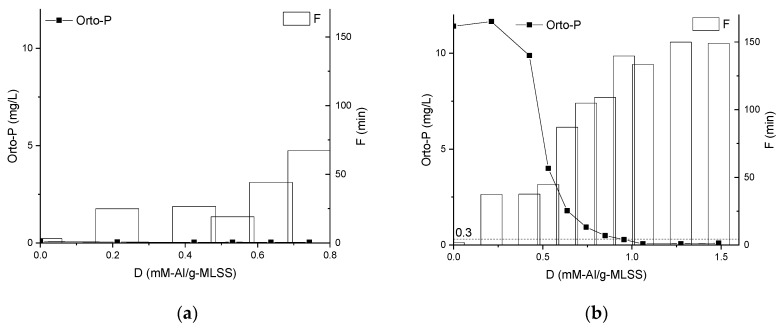
Influence of chemical enhancer dose on the concentration of orthophosphates in filtrate and filtration duration of Mixed Liquors from MBBR (**a**) and IFAS (**b**).

Dosing of the chemical enhancer did not affect the concentration of orthophosphates in the MBBR ML (Figure 7) due to pre-coagulation at the wastewater treatment plant that already achieved a low level of orthophosphates (<0.1 mg/L). The desired concentration of orthophosphates (<0.3 mg/L) in the IFAS ML was reached at dose 1.0 mM-Al/g-MLSS (Figure 3a).

### 3.3. Properties of the Sediments

**Figure 8:**
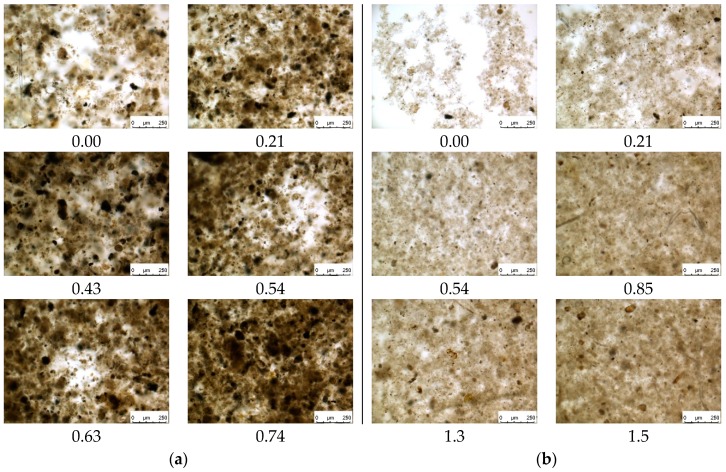
Light microscopy of sediments after Total Recycle Tests with Mixed Liquors from MBBR (**a**) and IFAS (**b**) at different doses of the chemical enhancer.

A clear difference can be observed from the light microscopy pictures of sediments after TRTs (Figure 8). In general, sediments formed from the MBBR ML are denser and more saturated, comparing with sediments from the IFAS ML. It is also visible that flock’s density is increasing with higher dosing of the coagulant.

**Figure 9:**
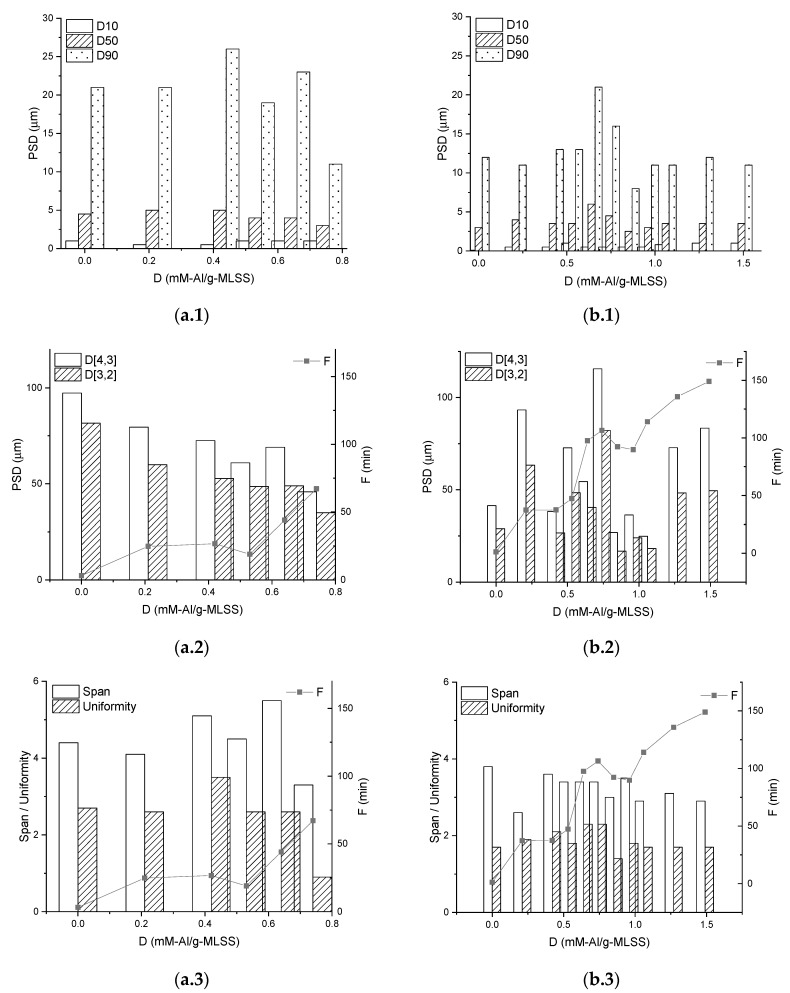
Particle Size Distribution in sediments after Total Recycle Tests with Mixed Liquors from MBBR (**a**) and IFAS (**b**) at different doses of the chemical enhancer, expressed as distribution points (**1**), means (**2**), span and uniformity (**3**).

Particle Sized Distribution (PSD) analysis carried out on selected microscopic images (Figure 9) did not show a pronounced tendency between PSD parameters and membrane filtration performance indicators. However, it can be noted that dosing of the chemical enhancer does not affect PSD parameters of the MBBR ML, but slightly an irregularly affects IFAS ML.

**Figure 10:**
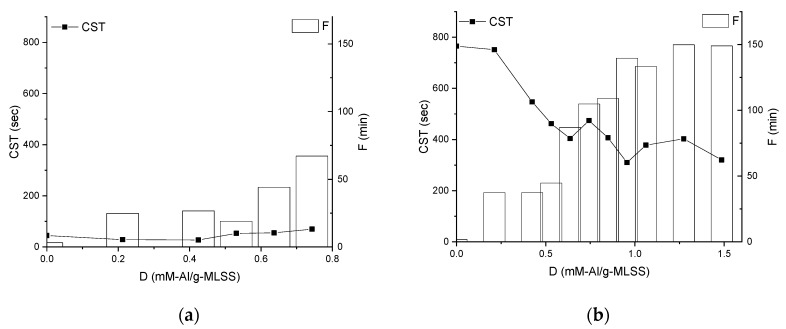
Influence of chemical enhancer dose on Capillary Suction Time of sediments and filtration duration of Mixed Liquors from MBBR (**a**) and IFAS (**b**).

While almost no change was observed on the Capillary Suction Time (CST) profile of the sediments from the MBBR ML (Figure 10a), CST of the sediments from IFAS declines pronouncedly from 750 to 310 sec, correlating with the increase of the filtration duration (Figure 10b).

### 3.4. Partial Least Squares Regression Analysis

**Figure 11:**
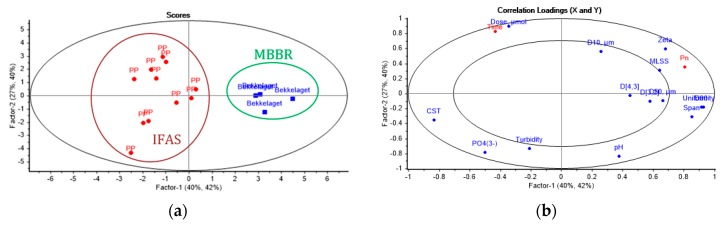
Scores plot (**a**) and correlation loadings plot (**b**) of the effects of chemical enhancer in Total Recycle Tests with Mixed Liquors from MBBR and IFAS.

**Figure 12:**
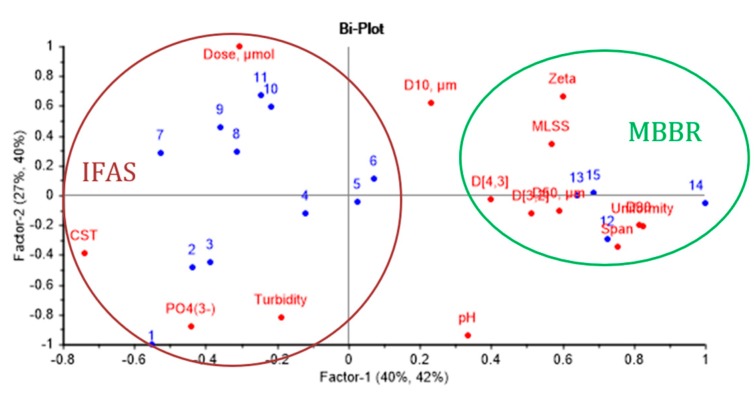
Bi-plot of effects of chemical enhancer in Total Recycle Tests with Mixed Liquors from MBBR and IFAS.

**Figure 13:**
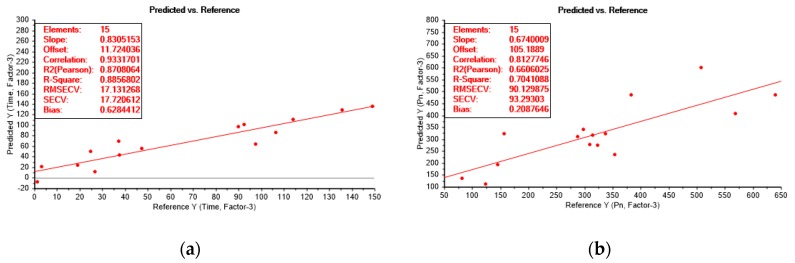
Prediction of filtration duration (**a**) and membrane permeability (**b**).

The obtained experimental results were used for modelling of chemical enhancement of MBBR and IFAS MLs in membrane filtration. Full cross-validation was applied to the derived PLS model, including Particle Size Distribution analysis of the sediments. The results of the partial least squares regression analysis (PLSR/PLS) based on data from the Total Recycle Tests (TRTs) are presented as scores plot (Figure 11a), loadings plot (Figure 11b), Bi-plot (Figure 12), prediction and reference plots (Figure 13).

The scores plot (Figure 11a) shows pronounced grouping of the results obtained for the MBBR and IFAS Mixed Liquors (MLs), which can be predominantly explained by Factor-1, while the separation of the classes along the Factor-2 and other factors is not apparent.

According to the correlation loadings plot (Figure 11b), the first two factors (latent variables) in total described 67% and 82% of the variance in the dataset, for X and Y respectively.

Further analysis of the loadings plot (Figure 11b) shows that Factor-1 clearly describes the parameters of the particle size distribution (PSD), i.e. Span, Uniformity, D90, D50, D[4,3], D[3,2], as well as MLSS, Zeta, PO_4_^3−^, CST, and permeability. Factor-2 accounts for Turbidity, Dose, pH D10 and the filtration time. The PSD indicators negatively affect CST, PO_4_3^−^ and Turbidity while they are positively correlated with the normalized permeability. Zeta potential and MLSS correlate positively with the normalized permeability, while pH negatively affects Dose and the filtration time. Most of the included variables are significant, while D50, D[4,3], D[3,2] lay in the inner ellipse and explain up to 50% of the variance in the dataset, which indicates that they are not important. However, it was decided to keep all the variables in the model to make it more reliable.

The Bi-plot (Figure 12) allows identifying significant variable for each data cluster. Samples from the IFAS plant are characterized by high levels of orthophosphates, turbidity, CST, and coagulant dosage, while the samples from the MBBR plant have high values of PSD parameters, such as span, uniformity, D50, D90, D[4,3], D[3,2]; MLSS, and zeta potential. It is worth noting that the latter group of samples were pre-coagulated at the wastewater treatment plant, resulting in higher zeta potential. Thus, the dosage required to reach the neutralization of the system, in this case, was lower than for the pilot plant samples, which merely contained the activated sludge.

The explained variances were computed for the model applying a different number of factors. Three factors provided the highest explanation of the total variation in Y by the model, which was equal to 79.5%.

The derived model had two responses (Y)—filtration time and permeability. The prediction of the filtration time resulted in the 0.89% validation Y variance explained by three factors, and the prediction of the normalized permeability led to the 0.70% validation Y variance explained by three factors (Figure 13). In both cases, R^2^ (Pearson) is close to R^2^ correlation—0.87 vs. 0.93 (Time) and 0.66 vs. 0.81 (P_n_), which indicates the reliability of the model. The slopes 0.83 (Time) and 0.67 (P_n_) indicated a good fit of the model to the majority of data. Root Mean Square Error of Cross Validation—(RMSEV) and the standard error of cross-validation (SECV) were around 17 for the prediction of the filtration time and were equal to 90 and 93, respectively, in the case of using normalized permeability as a response function. The prediction of the filtration time, as well as permeability, exhibited low values of the bias: 0.6 and 0.2 respectively. To sum up, the derived model is reliable and can be used for future predictions for the defined number of factors.

## 4. Discussion

Confirming general improvement of membrane separation performance reported in other studies on chemical enhancement in Bf-MBR with iron an pre-polymerized aluminium coagulants [27,28], the obtained results demonstrate a possibility to achieve lower membrane fouling rates with dosing of aluminium sulphate coagulant into MBBR and IFAS mixed liquors by extending operational cycles (F) by 20 and 80 time respectively (Figure 3a) as well as increasing operating permeability (P_n_) of membrane separation (Figure 3b). With a tendency of their product F × P_n_ to increase (Figure 3c), the consumption of additional chemicals and dosing control measures can be justified for chemical enhancement for retrofitting IFAS systems into MBR.

Taking into account that 0.7 mM-Al/g-MLSS of coagulant was added to the MBBR ML at the wastewater treatment plant before the TRTs, the difference observed in the permeability profiles (Figure 3b) can be explained by the absence of particles similar by diameter to membrane pore size due to pre-coagulation. This is well agreed with the studies by other researchers [19] and confirmed later by the results of light microscopy (Figure 8). In the case of IFAS ML, relatively stable permeability is an indication that reversible fouling dominates within a short series of TRTs with backwashes after each test.

A narrow range of the chemical enhancer concentration points to the destabilization of suspension, typical for coagulation. It also can be noticed that the IFAS curve approaches the saturation limit at the dosing >1.0 mM-Al/g-MLSS. This could be due to occurrence of complete colloid destabilization below 1.0 mM-Al/g-MLSS and no further influence of additional dosing over the ionic strength that is sufficient to compress the diffuse part of the double layer [42].

Measurement of pH during dosing of the chemical enhancer (Figure 4) indicates that stepwise hydrolysis of aluminium sulphate takes place both in MBBR and IFAS MLs. It can be expected that during the transition from the free aqua metal ion to the insoluble metal hydroxide precipitate, a series of hydrolytic reactions take place [42] affecting the MLs as also confirmed by the measurements of electrokinetic potential (Figure 5).

With a dispersion destabilized by hydrolyzed aluminium sulphate, if an excess of coagulant is applied, and the suspension is made sufficiently acidic, restabilization occurs as evidenced by reversion of particle surface charge back to the original value [42], identified by electrokinetic measurements in the region above 0.4 mM-Al/g-MLSS in MBBR ML (Figure 5a). This effect is also visible in Figure 6a as an increase of turbidity in the MBBR ML, however, it does not affect filtration duration. The reason for this can be that charge repulsion between foulants and membrane surface play a greater role than other factors like particle concentration and size as also observed by [28]. This effect is not observed for the IFAS ML in the whole range of dosing up to 1.5 mM-Al/g-MLSS probably due to higher concentration and different nature of foulants.

As metal coagulants have a pronounced tendency to polymerize during hydrolysis reactions [41], further addition of the chemical enhancer improves consolidation of sediments and leads to the formation of larger flocks and particles as progressively visible on the microscopic images (Figure 8).

Decreasing CST (Figure 10b) can be interpreted as an indication of charge compensation in the sludge flocks by the chemical enhancer and release of electrostatically bonded water. From the practical point of view, this also means better dewaterability of sediments during post-treatment of excess sludge.

## 5. Conclusions

In response to the growing environmental concerns and demands for wastewater reuse, Membrane Bioreactor can provide technological opportunities for retrofitting of existing MBBR and IFAS wastewater treatment plants. In such cases, chemical enhancement with aluminium sulphate can help to achieve lower membrane fouling rates by extending operational cycles up to 80 times and increasing operating permeability of membrane separation by 1.3 times for IFAS.

To secure the positive influence of the chemical enhancer on MBR performance, it is necessary to control its dosing, preventing acidification and restabilization of mixed liquor suspension.

It has been found that charge neutralization is the dominating mechanism of aluminium sulphate action as a chemical enhancer in Bf-MBR based on MBBR and IFAS mixed liquors; however, properties of the membrane surface influencing charge repulsion of foulants should be considered together with the secondary ability of the coagulant to improve consolidation of sediments.

Further research is needed to validate the potential application of various chemical enhancers, e.g., metal-based and organic coagulants, polymers, and proof the concept both analytically and experimentally.

## Figures and Tables

**Table 1:** Characteristics of Mixed Liquors (MLs) sampled from MBBR and IFAS treatment plants.

Parameters	ML Type	
	MBBR	IFAS
MLSS, g/L	4.72	3.53
Turbidity, NTU	29.7	91.1
pH	7.3–7.5	6.6–7.0
Zeta, mV	−8.2	−14.2
CST, sec.	45	765
PO_4_^3−^, mg/L	0.07	11.4
Viscosity, mPa/s	1.11	1.05

**Table 2:** Partial Least Squares regression parameters.

Predictors (X)	Designation
Coagulant dosage, mM-Al/g-MLSS	D
Capillary Suction Time of sediments, sec	CST
Supernatant turbidity, NTU	Turb
Supernatant zeta potential, mV	Zeta
Mixed Liquor Suspended Solids, g/L	MLSS
Orthophosphates in permeate, mg/L	PO_4_^3−^
Supernatant pH	pH
**Particle Size Analysis**	
10%, 50% 90% distribution points	D10, D50, D90
Surface Area Moment Mean	D[3,2]
Volume Moment Mean	D[4,3]
Span	Span
Uniformity	Uniformity
**Responses (Y)**	
Average permeability, L/m^2^⋅h⋅bar	P_n_
Filtration duration	F
